# Neural networks and the anti-inflammatory effect of transcutaneous auricular vagus nerve stimulation in depression

**DOI:** 10.1186/s12974-020-01732-5

**Published:** 2020-02-12

**Authors:** Chun-Hong Liu, Ming-Hao Yang, Guang-Zhong Zhang, Xiao-Xu Wang, Bin Li, Meng Li, Marie Woelfer, Martin Walter, Lihong Wang

**Affiliations:** 1 Beijing Hospital of Traditional Chinese Medicine, Capital Medical University, Beijing, 100010 China; 2 Beijing Institute of Traditional Chinese Medicine, Beijing, 100010 China; 3 Dermatological Department, Beijing Hospital of Traditional Chinese Medicine, Capital Medical University, Beijing, 100010 China; 4 Acupuncture and Moxibustion Department, Beijing Hospital of Traditional Chinese Medicine, Beijing Key Laboratory of Acupuncture Neuromodulation, Capital Medical University, Beijing, 100010 China; 5 Clinical Affective Neuroimaging Laboratory (CANLAB), Otto-von-Guericke-University Magdeburg, Magdeburg, 39120 Germany; 6 Department of Psychiatry and Psychotherapy, Jena University Hospital, Jena, 07743 Germany; 7 Department of Biomedical Engineering, New Jersey Institute of Technology, Newark, NJ 07102 USA; 8 Department of Behavioral Neurology, Leibniz Institute for Neurobiology, Magdeburg, 39118 Germany; 9 Center of Behavioral Brain Sciences, Otto-von-Guericke University, Magdeburg, 39118 Germany; 10 Department of Psychiatry, University of Connecticut Health Center, Farmington, CT 06030 USA

**Keywords:** Vagus nerve, Transcutaneous auricular vagus nerve stimulation, Depression, Brain network, Anti-inflammation

## Abstract

Transcutaneous auricular vagus nerve stimulation (taVNS) is a relatively non-invasive alternative treatment for patients suffering from major depressive disorder (MDD). It has been postulated that acupuncture may achieve its treatment effects on MDD through suppression of vagal nerve inflammatory responses. Our previous research established that taVNS significantly increases amygdala–dorsolateral prefrontal cortex connectivity, which is associated with a reduction in depression severity. However, the relationship between taVNS and the central/peripheral functional state of the immune system, as well as changes in brain neural circuits, have not as yet been elucidated. In the present paper, we outline the anatomic foundation of taVNS and emphasize that it significantly modulates the activity and connectivity of a wide range of neural networks, including the default mode network, executive network, and networks involved in emotional and reward circuits. In addition, we present the inflammatory mechanism of MDD and describe how taVNS inhibits central and peripheral inflammation, which is possibly related to the effectiveness of taVNS in reducing depression severity. Our review suggests a link between the suppression of inflammation and changes in brain regions/circuits post taVNS.

## Background

Major depressive disorder (MDD) is a common, costly, and potentially life-threatening psychiatric illness characterized by anhedonia, reduced energy, rumination, impaired cognition, vegetative symptoms, and suicidal tendency [1]. According to the “kindling theory,” subsequent episodes of MDD are correlated with a high number of previous episodes, even with milder stressors [2]. Individuals prone to recurrence may experience residual symptoms, including persistent subclinical depressive symptoms, rumination, impaired attentional control, and cognitive decline from the previous depressive episode [1, 3]. As a result, people with recurrent remitted MDD experience difficulty recovering from negative emotions and exhibit a persistent reduction in positive affect, resulting in a sustained depressed mood [4]. Thus, MDD treatment should aim for full recovery—that is, freedom from symptoms and a full restoration of social function at work [5]. Despite the possibility of its incurring skin irritation or redness, which is its most common side effect, “transcutaneous auricular vagus nerve stimulation” (hereafter, “taVNS”) is frequently used in the treatment of MDD, especially for residual symptoms [6].

The most widely used therapeutic alternatives for MDD are antidepressant medications, psychotherapy, cognitive behavioral therapy, deep-brain stimulation, electroconvulsive therapy, and repetitive transcranial magnetic stimulation [7]. However, the response rate of antidepressant medications is unsatisfying, and in up to 35% of patients, MDD remains recurrent and resistant to treatment [8]. In view of such facts, vagus nerve stimulation (VNS) was approved by the United States Food and Drug Administration in 2005 as an adjunctive long-term treatment for refractory MDD patients of 18 years of age or older who are not responsive to four or more antidepressant treatment trials [9]. Importantly, VNS has a demonstrated anti-inflammatory effect which might be a significant reason for its efficacy in patients who did not respond to antidepressants [7, 10]. However, this approach is limited by the potential side effects, including surgical complications, dyspnea, pharyngitis, pain and tightening in the larynx, and vocal strain [11, 12]. The auricular branch of the vagus nerve, also known as the Alderman’s nerve or Arnold’s nerve, innervates the external ear [13, 14], and the efficacy of auricular acupuncture and its antidepressive mechanism may be related to that found for VNS [15]. There is evidence that intermittent and chronic stimulation of the taVNS can greatly improve Hamilton Depression Rating Scale (HAM-D) scores without surgery, compared with the scores obtained in a sham taVNS group, and it is also considered to be highly practical and convenient owing to its strong safety and tolerability profile [16].

The theory behind taVNS postulates that the vagus nerve plays important roles in the relationship between the spleen, gut, brain, and inflammation [17]. It is believed that taVNS is linked to the microbiome–brain–gut axis, which regulates the relationship between brain regions mediating antidepressant effects (e.g., amygdala, ventral striatum, dorsal striatum, and ventromedial prefrontal cortex) and the gut connected with the splenic nerve, which is thought to reduce inflammation [18, 19]. Two meta-analyses have shown that the levels of proinflammatory cytokines, such as tumor necrosis factor-alpha (TNF-α), interleukin (IL)-6, IL-1, and C-reactive protein (CRP), are increased during depressive episodes [20, 21]. The findings of a recent review indicate that activation of immune–inflammatory pathways may affect monoaminergic and glutamatergic neurotransmission and contribute to MDD pathogenesis in at least a subset of patients [22]. Innate immune activation and inflammation have been reported to constitute a pathophysiologic mechanism in a subgroup of depressed patients with elevated inflammatory markers [23]. For example, increased plasma CRP was associated with reduced functional connectivity in a widely distributed network including the ventral striatum, parahippocampus, amygdala, orbitofrontal cortex, insula, and posterior cingulate cortex (PCC) [24], while plasma and cerebrospinal fluid CRP were associated with chemical shift imaging measures of basal ganglia glutamate in 50 medication-free MDD outpatients [25]. In another study, it was postulated that immune dysregulation or chronic inflammation might be present in recurrent remitted MDD [26]. Equally, other authors have found that the mechanism underlying taVNS treatment might be associated with persistent inhibition of neuroinflammatory sensitization [27]. However, taVNS-based biosignatures associated with inflammation-induced neural dysregulation in MDD have not been well characterized to date.

In the present review, we discuss the potential immunologic mechanisms and neuroimaging markers for taVNS treatment of MDD. First, we outline the history of auricular acupuncture. Then, we present the anatomic foundation of taVNS. Next, we focus on the relationship between brain regions or circuits and taVNS. Fourth, we address how taVNS inhibits central and peripheral inflammation, indicating a possible mechanism for its efficacy. Lastly, we describe an important link between taVNS and the microbiome–brain–gut axis.

## The history of auricular acupuncture

**Fig. 1:**
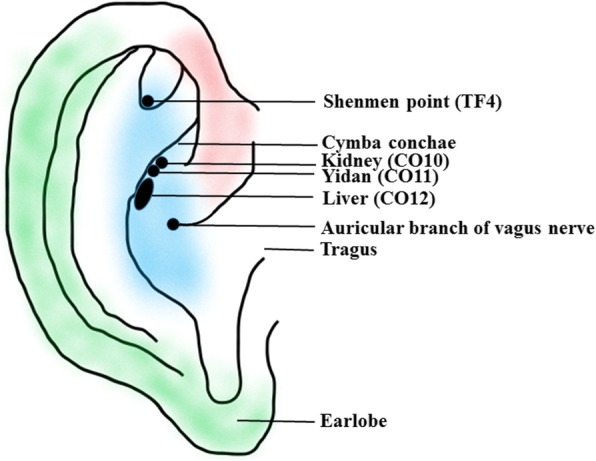
Innervation of the human auricle, including the auricular branch of the vagus nerve (blue shading); the black areas show the specific auricular acupoints. TF4 and CO10–12 are used to stimulate the auricular branch of the vagus nerve

Contemporary auricular acupuncture is part of traditional Chinese medicine that has recently attracted scientific and public attention as it becomes increasingly accessible to the general public in modern China [28] (see Fig. 1). According to writings dating back to the Chinese *Miraculous Pivot*, part of the *Huangdi Neijing* (*The Yellow Emperor’s Inner Canon*), and those of Hippocrates in the West [29], the ear is not isolated but rather is directly or indirectly connected with 12 meridians [30]. Since Dr. Paul Nogier, a French neurologist, created a map of the ear resembling an inverted fetus [31], auricular acupuncture has adopted a more systemic approach, and may serve as a source of alternative non-pharmacologic therapies for MDD. In 1990, the World Health Organization recognized auricular acupuncture as a microacupuncture system that can have a positive impact on regulating whole-body function [32]. By 2002, Peuker and Filler had described a branch of the vagus nerve distributed in the concha (including in the cymba conchae and cavum conchae) [33]. Having considered the anatomy of the neural pathways in the external auricle and their clinical and experimental findings relating to the mechanisms of taVNS, Usichenko et al. [34] proposed that the analgesic effects of auricular acupuncture could be explained by stimulation of the auricular branch of the vagus nerve [34]. Thus, it is very likely that taVNS is derived from the Chinese system of energy circulation along the meridians, which connect “diseased” body organs with the external auricle and explain the reflexotherapy effects of auricular acupuncture [35].

## The anatomic foundation of taVNS

The vascularization and innervation of the auricle constitute the theoretical basis of taVNS; thus, similar effects to those obtained with VNS may be achieved by superficially stimulating the area of the ear that has vagus nerve innervation [36]. Using 14 ears from seven German cadavers, Peuker and Filler found that four different nerves are distributed to the external ear, comprising the auriculotemporal nerve, the auricular branch of the vagus nerve, the lesser occipital nerve, and the greater auricular nerve [33]. In the context of the present study, at least, the most important nerve is the auricular branch of the vagus nerve, which supplies most of the area around the auditory meatus and cymba conchae [33]. Burger and Verkuil, however, suggested that the tragus of the auricle is not innervated by the auricular branch of the vagus nerve [37]. Currently, the universally accepted hypothesis relating to taVNS is that external somatosensory inputs interact with internal organ responses and the central neural networks [38].

The vagus nerve consists of 20% motor efferent and 80% sensory afferent fibers, which are important for relaying visceral, somatic, and taste sensations [39]. The brain receives information from the afferent projections of the vagus. The afferent fibers project to the nucleus tractus solitarius (NTS) and locus coeruleus (LC) in the brainstem [40] and then form direct and indirect ascending projections from the NTS to many areas of the brain (e.g., midbrain, hypothalamus, amygdala, hippocampus, and frontal lobe) [41, 42]. A recent systematic review has shown that both the autonomic and the central nervous systems can be modified by auricular vagal stimulation via projections from the auricular branch of the vagus nerve to the NTS [43]. Another review, by Kong et al. [28], showed that the auricular branch of the vagus nerve projects to the NTS, which is further connected with other brain regions, such as the LC, parabrachial area, hypothalamus, amygdala, anterior cingulate cortex, anterior insula, and nucleus accumbens [26]. Functional magnetic resonance imaging (fMRI) and taVNS at the posterior side of the left outer auditory canal have revealed that limbic deactivations are prominent in the area of the parahippocampal gyrus, PCC, and right thalamus [44]. Two fMRI studies carried out during taVNS at the inner side of the tragus or outer auditory canal in healthy subjects have also provided evidence of effectiveness in the generation of blood oxygenation level-dependent signal activations in the LC, nucleus accumbens, thalamus, prefrontal cortex, postcentral gyrus, PCC, and insula [45, 46].

In addition, the vagus nerve regulates the function of the autonomic nervous system from its efferent projections [15]. The vagus nerve runs from the brainstem through the neck to many peripheral organs, including the lungs, liver, stomach, intestines, and spleen [15, 47]. The vagus nerve system suppresses the release of proinflammatory cytokines such as TNF, IL-1β, IL-6, and IL-18 [48, 49]. The spleen is the largest secondary lymphoid organ and hosts a wide range of immunologic functions alongside its roles in the removal of older erythrocytes from the circulation and clearance of blood-borne microorganisms and cellular debris [50]. Given its diverse functions, the spleen allows for interactions between the circulation of immune cells, immune-mediated bacterial clearance, and immune reactivity [51]. Further, the vagus nerve provides extensive innervation to the gastrointestinal tract, where there are substantial depots of lymphoid tissue [52]. Currently, there is some debate concerning the most peripheral branch of the vagus nerve [53], which demonstrates that there are still several unanswered questions regarding the anatomic basis of taVNS [54].

## The inflammatory mechanism of MDD

Many biological hypotheses exist with respect to the etiology of MDD, including suppositions incorporating monoamine neurotransmitter disturbance, endocrine system dysfunction, decreased neurotrophic factors, and excessive proinflammatory cytokines in MDD [55]. Among them, inflammatory mechanisms have attracted increased attention, and the inflammatory processes have been found to play an important role in the pathophysiology for at least a subgroup of individuals with MDD [22]. A diverse array of evidence has been reported regarding increased plasma cytokines due to both peripheral chronic inflammation and central microglial activation involved in the pathophysiology of MDD [56]. The relationship between MDD and inflammation is bidirectional, with one predisposing the other [57]. Peripheral stimuli such as chronic infection or stress may inhibit the negative feedback of the hypothalamic−pituitary−adrenal (HPA) axis, trigger the activation of microglia in the brain, and increase the permeability of the blood–brain barrier, resulting in excessive activation of proinflammatory cytokines [26, 58]. On the other hand, increased proinflammatory cytokines may cause MDD by activating the HPA axis, which results in a depletion of serotonin with an increased activity of the indoleamine-2,3-dioxygenase (IDO) enzyme in the tryptophan–kynurenine system [59]. Studies with animal models as well as clinical research have identified increased plasma inflammatory markers, such as IL-1, IL-2, IL-6, and TNF-α [60]. In some depression cases, chronic inflammation or immune dysregulation has been found to play an essential role in the onset and maintenance of recurrent and refractory MDD [22, 26, 61]. There is a wealth of evidence from randomized control trials suggesting that anti-inflammatory agents are superior to placebos as an add-on therapy and as a monotherapy in MDD patients [62]. These findings on the involvement of low-grade chronic inflammation in the etiopathogenesis of MDD provide further empirical support for the argument that special treatment is needed for subtypes of MDD associated with inflammation.

## Relationships between microbiota, MDD, and VNS

The microbiota is a collection of trillions of microorganisms, including 1014 bacteria [63], that is involved in energy harvesting from the breakdown of indigestible food substances, micronutrient absorption, immune system stimulation, neurologically active substance production (e.g., gamma-aminobutyric acid (GABA) and short-chain fatty acids), and HPA axis regulation [64]. Gut microbiota may impact on MDD through a variety of mechanisms, such as the satiety and reward circuits, the HPA axis, immunomodulation, the metabolism of tryptophan, and the production of various neuroactive compounds [64, 65]. Recent work has shown that serum concentrations of immunoglobulin A and immunoglobulin M levels directed against the gut bacteria (i.e., Hafnia alvei, *Pseudomonas aeruginosa*) were significantly higher in MDD patients than in healthy controls [66]. Moreover, probiotic interventional studies offer supportive evidence, in that psychobiotics containing *Lactobacillus acidophilus*, *Lactobacillus casei*, and *Bifidobacterium bifidum* have been found to have the ability to improve depressive symptoms in MDD patients [67]. A clinical study has revealed that gut microbiotic compositions such as Firmicutes, Actinobacteria, and Bacteroidetes were significantly different between MDD patients and healthy controls [68]. In addition, fecal microbiota transplantation from MDD patients into mice has been shown to result in depression-like behaviors [68]. Changes in the overall gut microbiota are relevant to mood states because gut microbiota interact with the brain via the HPA axis or the vagus nerve pathways [69]. Approximately 80% of vagus nerve fibers are afferent and relay signals from the brain to the viscera, including the digestive tract [70]. Microbiota may also indirectly result in MDD through the mediation of the levels of neurotransmitters such as serotonin, noradrenalin, dopamine, and GABA [71].

## Neuroimaging biomarkers related to taVNS treatment in healthy participants

To date, six studies have used fMRI to investigate the brain response to taVNS in healthy participants (14, 44–46, 54, 72; see Table 1). Stimulation of the inner tragus and cymba conchae revealed activation of the NTS and the LC, a brainstem nucleus that receives direct input from the tractus solitarius. Stimulation at the inferoposterior wall of the auditory canal revealed the weakest activation of these two nuclei [72]. Using stimulation at the left outer auditory canal, Kraus et al. [46] found increased activation in the insula, precentral gyrus, and thalamus, as well as decreased activation in the amygdala, hippocampus, parahippocampal gyrus, and middle and superior temporal gyrus; stimulation of the posterior wall, however, lead to activation of the tractus solitarius [46]. Using stimulation at the anterior left auditory canal, Kraus et al. [44] found decreased activation in the parahippocampal gyrus, PCC, and right thalamus (pulvinar), and decreased activation in the NTS and LC [44]. Using stimulation of the left inner tragus, Dietrich et al. [45] found increased activation in the left LC, thalamus, left prefrontal cortex, right and left postcentral gyrus, left posterior cingulate gyrus, and left insula, as well as decreased activation in the right nucleus accumbens and right cerebellar hemisphere [45]. Using either left tragus (active) or earlobe (control) stimulation, Badran et al. [54] found increased activation in the contralateral postcentral gyrus, bilateral insula, frontal cortex, right operculum, and left cerebellum in active stimulation and increased activation in the right caudate, bilateral anterior cingulate, cerebellum, left prefrontal cortex, and middle cingulate with the active stimulation versus control stimulation [54]. Furthermore, increased activation was found in the ipsilateral NTS, bilateral spinal trigeminal nucleus, dorsal raphe, LC, contralateral parabrachial area, amygdala, nucleus accumbens, and bilateral paracentral lobule, as well as decreased activation in the bilateral hippocampus and hypothalamus after stimulation at the cymba conchae [14]. In summary, these functional neuroimaging studies of the mechanism of taVNS in healthy participants confirmed the involvement of the NTS and the LC, two structures that are highly associated with the vagus nerve [14, 45], and showed a change in the limbic structures involved in depression-related neural circuits [44, 73, 74].

**Table 1:** Prior research—stimulated areas and activated brain regions studied

Study	Stimulated area	Activated brain regions^a^
Yakunina et al. (2017) [72]	The inner tragus and cymba conchae and the inferior posterior wall of the auditory canal	The NTS and the LC
Kraus et al. (2007) [46]	The left outer auditory canal	Increased activation in the insula, precentral gyrus, and thalamus; decreased activation in the amygdala, hippocampus, parahippocampal gyrus, and middle and superior temporal gyrus
	The posterior wall	The NTS
Kraus et al. (2013) [44]	The anterior left auditory canal	The parahippocampal gyrus, PCC, and right thalamus (pulvinar), NTS, and LC
Dietrich et al. (2008) [45]	The left inner tragus	The left LC, thalamus, left prefrontal cortex, right and left postcentral gyrus, left posterior cingulate gyrus, and left insula, as well as decreased activation in the right nucleus accumbens and right cerebellar hemisphere
Badran et al. (2018) [54]	The left tragus (active) or earlobe (control)	The contralateral postcentral gyrus, bilateral insula, frontal cortex, right operculum, left cerebellum and the right caudate, bilateral anterior cingulate, cerebellum, left prefrontal cortex, and middle cingulate
Frangos et al. (2015) [14]	The cymba conchae	Increased activation in the ipsilateral NTS, bilateral spinal trigeminal nucleus, dorsal raphe, LC, contralateral parabrachial area, amygdala, nucleus accumbens, bilateral paracentral lobule; decreased activation in the bilateral hippocampus and hypothalamus

*LC* locus coeruleus, *NTS* nucleus tractus solitaries, *PCC* posterior cingulate cortex

^a^In healthy participants

**Fig. 2:**
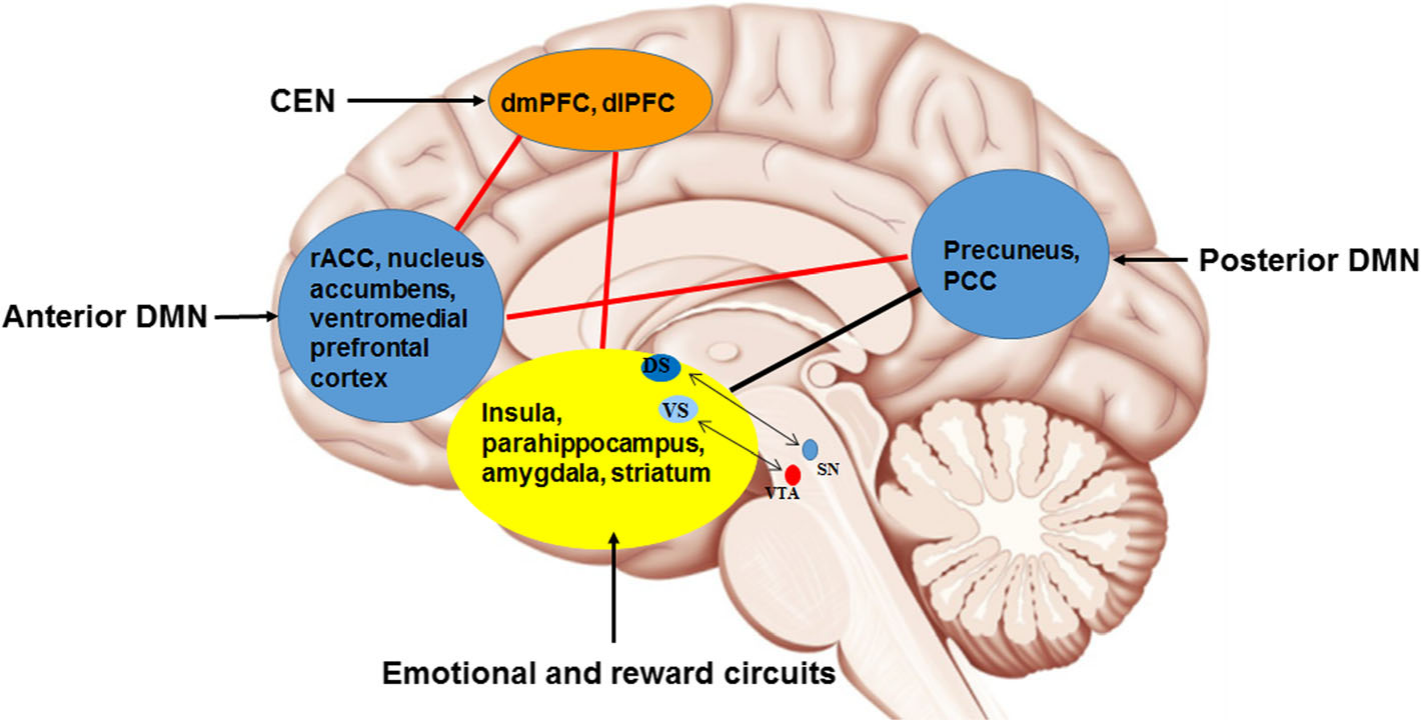
Proposed model of the mechanism of taVNS in the central nervous system (schematic). Anterior DMN = anterior default mode network; CEN = central executive network; dlPFC = dorsolateral prefrontal cortex; dmPFC = dorsomedial prefrontal cortex; DS = dorsal striatum; LC = locus coeruleus; mPFC = medial prefrontal cortex; NTS = nucleus tractus solitarius; PCC = posterior cingulate cortex; posterior DMN = posterior default mode network; rACC = rostral anterior cingulate cortex; VS = ventral striatum.

In addition to the neuroimaging findings in healthy participants, taVNS has also been studied in relation to MDD (see Table 2). Using fMRI and mega-press ^1^H-magnetic resonance spectroscopy, Li et al. [75] found increased functional connectivity (FC) between the left rostral anterior cingulate cortex (rACC) and a set of regions including the bilateral precuneus, bilateral insula, right dorsolateral prefrontal cortex (dlPFC), left anterior cingulate cortex, and left middle cingulate cortex, and between the right rACC and left lingual gyrus, but decreased neurotransmitter concentrations of GABA and glutamate in treatment-resistant MDD patients receiving taVNS and sertraline for 8 weeks [75]. Analyzing the hypothalamic subregion FC of 41 mild to moderate MDD patients, Tu et al. [76] found decreased FC between the bilateral medial hypothalamus and rACC in the taVNS group but not in the sham taVNS group. Furthermore, the strength of this FC was significantly correlated with HAM-D improvements after 4 weeks of taVNS [76]. Studying the nucleus accumbens FC of 41 MDD patients receiving continuous real or sham taVNS for 4 weeks, Wang et al. [77] found increased FC between the left nucleus accumbens and bilateral medial prefrontal cortex (mPFC)/rACC, and between the right nucleus accumbens and left insula, occipital gyrus, and right lingual/fusiform gyrus in the taVNS group, compared with the sham taVNS group; the strength of FC between the left nucleus accumbens and bilateral mPFC/rACC was negatively associated with the HAM-D score changes in the taVNS group after 1 month of treatment in the taVNS group, but not in the sham group [77]. Furthermore, decreased FC between the default mode network (DMN) and anterior insula and parahippocampus, and increased FC between the DMN and precuneus and orbital prefrontal cortex have demonstrated in the taVNS group, compared with sham taVNS group; the strength of the increased FC was also associated with improvements in HAM-D scores using the DMN connectivity in MDD [78]. Further, the fMRI signal in the left anterior insula was increased by taVNS, compared with sham taVNS, and the insula activation level was associated with HAM-D improvement in longitudinal 4-week treatment outcomes [79]. Using amygdala resting-state FC changes at baseline and after 4 weeks of taVNS and sham taVNS treatments, our research team reported that there was increased FC between the right amygdala and left dlPFC in the taVNS group, compared with the sham taVNS group; the strength of the increased FC was also associated with HAM-D score reduction, as well as decreases on the anxiety and retardation HAM-D subscales [36]. Taken together, these findings demonstrate that taVNS produces changes in resting-state nodes distributed throughout a wide range of neural networks, including the DMN, salience network (SN) (insula, mPFC/rACC, and parahippocampus), central executive network (CEN) (dlPFC), and reward circuits (orbital prefrontal cortex). A review by Mulders et al. [80] has highlighted an increased FC between the anterior DMN and the SN, an increased FC within the anterior DMN, and a decreased FC between the posterior DMN and the CEN in MDD [80]. Following the work of Mulders et al. [80], in the present study, we propose a model (Fig. 2) focusing on taVNS: decreased FC between the posterior DMN and emotional and reward circuits and increased FC between the anterior and posterior DMN, between the anterior DMN and CEN, and between the CEN and emotional and reward circuits might be more specific to taVNS.

**Table 2:** Clinical and neuroimaging findings relating to taVNS treatment in MDD

Study	Characteristics of MDD samples	MDD group		Brain regions	Method
		Real taVNS	Sham taVNS		
Li et al. (2019) [75]	Treatment-resistant MDD	1	0	Increased connectivity between rACC and bilateral precuneus, bilateral insula, right dlPFC, left anterior cingulate cortex, left middle cingulate cortex	FC with rACC as seed
Tu et al. (2018) [76]	Mild to moderate MDD	41		Decreased connectivity between bilateral medial hypothalamus and rACC	FC with hypothalamic subregion as seed
Wang et al. (2017) [77]	Mild to moderate MDD	41		Increased FC between left nucleus accumbens and bilateral mPFC/rACC, and between right nucleus accumbens and left insula, occipital gyrus, and right lingual/fusiform gyrus	FC with nucleus accumbens as seed
Fang et al. (2016) [78]	MDD	25		Decreased FC between DMN and anterior insula and parahippocampus, and increased FC between DMN and precuneus and orbital prefrontal cortex	Independent component analysis
Fang et al. (2016) [79]	MDD patients	17	21	fMRI signal increases in the left anterior insula	Task fMRI with taVNS or sham taVNS
Liu et al. (2016) [36]	active and remitted MDD	28	25	Increased FC between right amygdala and left dlPFC	FC with right amygdala as seed

*dlPFC* dorsolateral prefrontal cortex, *DMN* default mode network, *FC* functional connectivity, *MDD* major depressive disorder, *mPFC* medial prefrontal cortex, *rACC* rostral anterior cingulate cortex, *taVNS* transcutaneous auricular vagus nerve stimulation

## taVNS and the inhibition of central and peripheral inflammation in MDD

Evidence has shown that only specific subpopulations of depressed patients may have an underlying immune dysregulation that could explain depression relapse and lack of therapeutic benefits of antidepressants [22, 81]. Stimuli such as inflammatory, infectious, and stressful challenges might trigger the activation of immune cells in the blood and peripheral tissues, and induce glial cells in the central nervous system to release proinflammatory cytokines [82]. Moreover, peripheral proinflammatory cytokines can reach the brain through leaky regions in the blood–brain barrier, cytokine signaling molecules (including p38 mitogen-activated protein kinase, nuclear factor kappa-light-chain-enhancer of activated B cells, signal transducer and activator of transcription 1a, and cyclooxygenase-2), activation of endothelial cells lining the cerebral vasculature, and binding to cytokine receptors associated with peripheral afferent nerve fibers (e.g., vagus nerve) [83, 84]. Central immune activation (e.g., macrophage accumulation and microglial activation) can affect the levels of acetylcholine through alpha-7 nicotinic acetylcholine receptors (α7 nAChRs) and produce anti-inflammatory effects [85]. During the eradication of invading microorganisms and removal of debris, the activation of α7 nAChRs alters the phenotype from M1-like (activated for antimicrobial activity) to M2-like (resolution, removal of debris) [86] in both peripheral and central macrophages [87]. Wang and colleagues have reported that the α7 nAChR subunit is essential for inhibiting cytokine synthesis by the cholinergic anti-inflammatory pathway (CAP) [88]. Tracey observed that the α7 nAChR induced the cholinergic inflammatory reflex, whereby inflammatory mediators (e.g., cytokines) in peripheral tissues activate the central nervous system via vagal afferents [89]; this, in turn, inhibits proinflammatory cytokine production and protects against systemic inflammation via the CAP that vagus nerve-released acetylcholine inhibits TNF-α release [90] or the connections of the vagus nerve with the spleen [91]. The distal end of the splenic nerve releases norepinephrine, which inhibits the release of TNF-α by spleen macrophages through binding to the β2 adrenergic receptor of spleen lymphocytes that release ACh [92]. Recent review studies have also indicated both peripheral and central anti-inflammatory effects in taVNS, exerted via α7 nAChRs [93].

VNS might have an anti-inflammatory effect on central serotonin levels and affect the HPA axis and cortisol levels [94]. In inflammation, proinflammatory cytokines such as IL-1 and TNF-α increase the activity of IDO [82, 95]. IDO decreases the synthesis of serotonin by catalyzing tryptophan through the production of kynurenic acid, quinolinic acid, and nicotinamide adenine dinucleotide [96, 97]. The depletion of serotonin results in the development of depressive symptoms, as suggested by the monoamine depletion hypothesis [59]. Another mechanism centers on a neuroendocrine pathway involving the HPA axis through a vagus pathway leading to the release of corticotrophin-releasing hormone, adrenocorticotropic hormone, and cortisol by acting directly on hypothalamic and pituitary cells [98, 99]. Thus, taVNS has anti-inflammatory properties both through its afferents (activating the HPA axis) and its efferents (via IDO), putting the vagus nerve at the interface of neurotransmitters, the neuroendocrine system, neuroinflammation, and immunity [100].

**Fig. 3:**
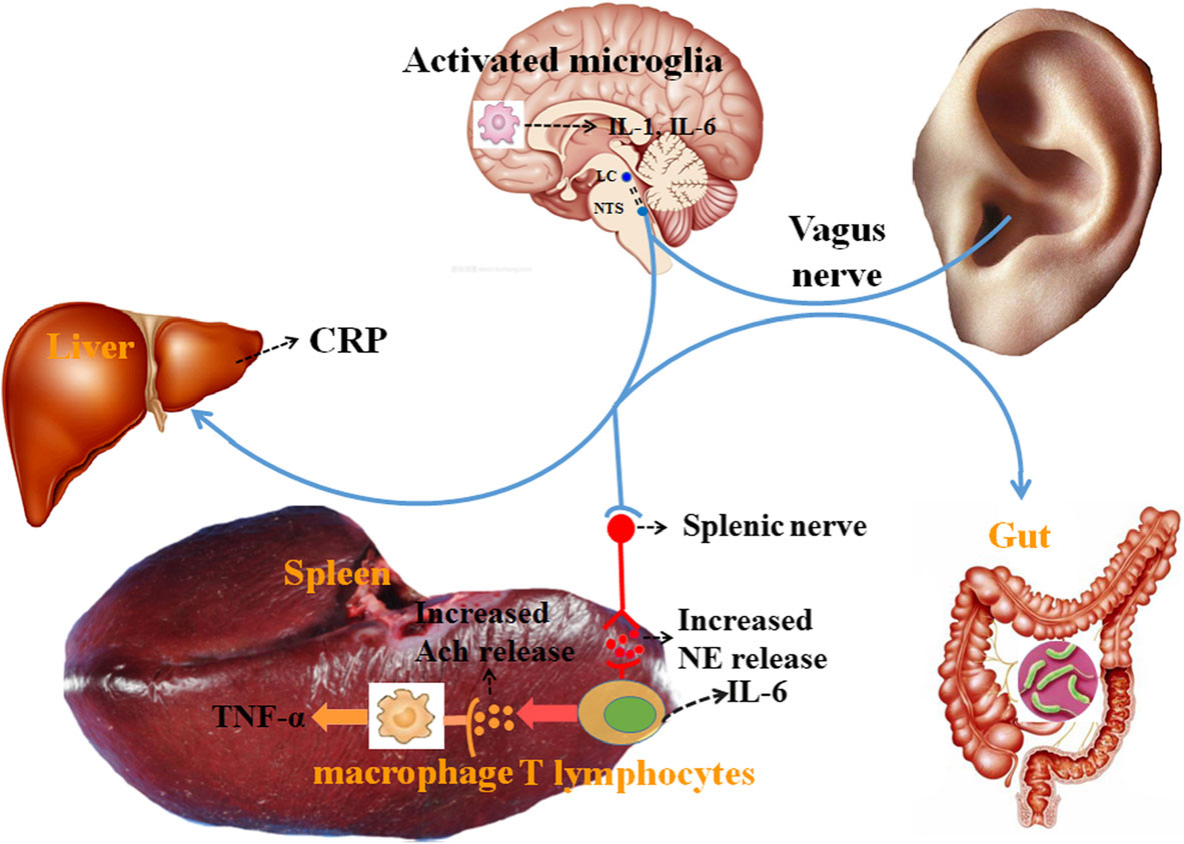
Hypothesized mechanisms of taVNS in the treatment of depression: direct and indirect modulation of the activity and connectivity of the key brain regions involved in depression, reducing neuroinflammatory sensitization and modulating the autonomic nervous system. ACh = acetylcholine; CRP = C-reactive protein; IL = interleukin; LC = locus coeruleus; NE = norepinephrine; NTS = nucleus tractus solitarius; TNF-α = tumor necrosis factor-α.

Generally, the CAP has an anti-TNF effect exerted by the vagus nerve, which dampens peripheral inflammation and decreases intestinal permeability, thus likely modulating microbiota composition [101]. Moreover, the vagus nerve establishes connections between the brain and the gut and transmits information about the state of the gastrointestinal tract to the brain via afferent fibers [102]. However, the vagus nerve does not directly interact with resident macrophages in the gut; hence, the exact nature of the anatomic interaction between the vagus nerve and the intestinal immune system is still a matter of debate [100]. Recent evidence supports the idea that the central nervous system interacts dynamically with the intestinal immune system via the vagus nerve to modulate inflammation through the HPA axis, IDO, and the CAP [101, 102]. The gut is an important control center of the immune system, in which immune cells are constantly in contact with the external environment, which includes food antigens, nutrients, and potential pathogens [103]. Taking into account the extensive innervation of the gastrointestinal tract, it is not surprising that the vagus nerve appears to play a role in modulating immune activation in the gut wall [104]. The vagus nerve senses microbiota metabolites through its afferents and generates an adaptive response in the regulation of gastrointestinal motility, acid secretion, food intake, and satiety [105]. As a result, taVNS represents a potential treatment for gastrointestinal and psychiatric disorders such as inflammatory bowel disease and MDD [83, 99]. Lim and colleagues found that acupuncture may achieve its treatment effects through vagal nerve–induced anti-inflammatory responses in internal organs [106]. Experimental evidence has suggested that taVNS could decrease the serum proinflammatory cytokines levels, such as TNF-a, IL-1β, and IL-6, as well as the proinflammatory transcription factor; for example, NF-kappa B p65 in endotoxemia was found to affect anesthetized rats [107]. Clinical evidence has suggested that VNS is associated with the abnormal profile of proinflammatory cytokines, such as IL-6, TNF-α, and TGF-β concentrations, in treatment-resistant MDD [108]. Such stimulation might have an anti-inflammatory effect on central serotonin levels and affect the HPA axis and cortisol levels [98]. Activation of the vagus nerve may modulate the neuroimmune system, the neuroendocrine system, and brain regions within the DMN, SN, and CEN (which are the “hotspots” involved in MDD). Therefore, we propose a model focusing on taVNS that can act on three pathways that may treat MDD: (1) regulation of the brain–gut axis through activation of the HPA axis; (2) inhibition of TNF-α release by macrophages through the CAP; (3) direct and indirect modulation of the activity of, and connectivity between, the DMN, SN, and reward circuits. The various mechanisms by which taVNS may improve depressive symptoms are illustrated in Fig. 3.

## Conclusions

In summary, we posit that taVNS can significantly reduce the symptoms of depression, such as anxiety, cognitive impairment, sleep disturbance, and feelings of hopelessness. Inflammation interacts with brain circuits via complicated direct and indirect pathways, including neuronal, immune-mediated, and neuroendocrine-mediated signaling. Of note, alterations within and between the DMN, SN, and CEN are “hotspots” involved in MDD, as reported in numerous imaging studies. taVNS can directly and indirectly decrease connectivity between the posterior DMN and emotional and reward circuits and increase connectivity between the anterior and posterior DMN, between the anterior DMN and CEN, and between the CEN and emotional and reward circuits. We infer that taVNS has anti-inflammatory properties that are exerted through activation of the HPA axis, the CAP, and brain regions or circuits in MDD. Additional studies are needed to further clarify the mechanism of brain function regulation by inflammation in taVNS.

## Data Availability

Not applicable.

## Abbreviations

CAP: Cholinergic anti-inflammatory pathway
CEN: Central executive network
dlPFC: Dorsolateral prefrontal cortex
DMN: Default mode network
FC: Functional connectivity
fMRI: Functional magnetic resonance imaging
HAM-D: Hamilton depression rating scale
HPA: Hypothalamic–pituitary–adrenal
IDO: Indoleamine-2,3-dioxygenase
IL: Interleukin
LC: Locus coeruleus
MDD: Major depressive disorder
mPFC: Medial prefrontal cortex
NTS: Nucleus tractus solitarius
PCC: Posterior cingulate cortex
rACC: Rostral anterior cingulate cortex
SN: Salience network
taVNS: Transcutaneous auricular vagus nerve stimulation
TNF-α: Tumor necrosis factor-alpha
VNS: Vagus nerve stimulation
α7 nAChR: Alpha-7 nicotinic acetylcholine receptor
